# Component-Level Effects of Enhanced Recovery After Surgery Protocols on Postoperative Outcomes Across Surgical Specialties: A Systematic Review

**DOI:** 10.7759/cureus.107401

**Published:** 2026-04-20

**Authors:** Bilal Ahmad, Muhammad Bux, Ali Haider Bajwa, Basil Ahmed Ali Ali Elgohary, Alara Khan

**Affiliations:** 1 Urology, York and Scarborough Teaching Hospitals NHS Foundation Trust, York, GBR; 2 General Surgery, Jinnah Postgraduate Medical Center, Karachi, PAK; 3 General Surgery, Mymensingh Medical College, Mymensingh, BGD; 4 Cardiothoracic Surgery, Ain Shams University, Cairo, EGY; 5 General Surgery, Fatima Jinnah Medical University, Lahore, PAK

**Keywords:** early enteral nutrition, early mobilization, enhanced recovery after surgery, eras, fluid management, length of stay, multimodal analgesia, perioperative care, postoperative complications, postoperative outcomes

## Abstract

Enhanced recovery after surgery (ERAS) protocols have been widely adopted across surgical specialties to improve postoperative outcomes; however, the relative contribution of individual components within these multimodal pathways remains unclear. This systematic review aimed to evaluate the association between specific ERAS components and postoperative outcomes across diverse surgical settings. A comprehensive literature search was conducted in PubMed/MEDLINE, Scopus, and Web of Science for studies published between January 1, 2015, and December 31, 2025, in accordance with Preferred Reporting Items for Systematic Reviews and Meta-Analyses (PRISMA) 2020 guidelines. Observational cohort studies assessing ERAS protocols with reported compliance or component-level data and relevant postoperative outcomes were included. Data extraction focused on study characteristics, ERAS components, compliance measures, and clinical outcomes, and risk of bias was assessed using the Newcastle-Ottawa Scale. Eight studies comprising 4,959 patients across colorectal, hepatobiliary, pancreatic, thoracic, gynecologic, gastric, and head and neck surgeries were included. Higher ERAS compliance was consistently associated with reduced postoperative complications and shorter length of stay, demonstrating a dose-response relationship in most studies. Component-level analyses identified early mobilization, early enteral nutrition, perioperative fluid optimization, minimization of invasive tubes and catheters, and multimodal analgesia as the most consistently beneficial interventions. The timing of implementation, particularly within the early postoperative period, emerged as a critical determinant of outcomes. However, heterogeneity across studies and findings from a large multicenter cohort suggest that partial or inconsistent ERAS implementation may not confer significant benefit. Overall, while adherence to ERAS protocols is associated with improved postoperative recovery, a subset of key components appears to drive these effects across surgical specialties. These findings support a targeted, component-focused approach to ERAS implementation, emphasizing early postoperative interventions and high-fidelity execution, particularly in settings where full protocol adherence may be challenging.

## Introduction and background

Enhanced recovery after surgery (ERAS) represents a multidisciplinary, evidence-based approach to perioperative care designed to attenuate the physiological stress response to surgery and promote early recovery [1]. Since its initial development in colorectal surgery, ERAS has been progressively adopted across a wide range of surgical specialties, including hepatobiliary, pancreatic, thoracic, gynecologic, and head and neck procedures [2,3]. Core elements of ERAS pathways typically span the preoperative, intraoperative, and postoperative phases and include patient education, optimized analgesia, goal-directed fluid therapy, early mobilization, and early enteral nutrition. Collectively, these interventions aim to reduce postoperative complications, shorten hospital length of stay, and improve overall patient outcomes [4].

A substantial body of literature has demonstrated that ERAS protocols are associated with improved postoperative recovery and reduced healthcare utilization. Observational studies and meta-analyses consistently report reductions in length of stay and complication rates following ERAS implementation [5,6]. However, ERAS pathways are inherently complex and consist of numerous interdependent components, often exceeding twenty individual elements depending on the surgical specialty. As a result, the relative contribution of individual components to the overall effectiveness of ERAS remains incompletely understood. In clinical practice, adherence to ERAS protocols is variable, and full implementation may be challenging due to institutional, logistical, and patient-related factors [7].

Recent investigations have increasingly focused on the role of ERAS compliance, with higher adherence rates generally associated with improved outcomes. While these findings support the importance of protocol fidelity, they do not clarify whether all ERAS components contribute equally to recovery or whether certain elements exert a disproportionate effect [8,9]. Emerging evidence suggests that specific interventions, particularly those implemented in the early postoperative period, may be more strongly associated with favorable outcomes. At the same time, heterogeneous findings across studies, including those reporting limited benefit with partial ERAS implementation, highlight the complexity of translating protocol-based care into consistent clinical improvement.

Given these considerations, a more granular understanding of ERAS pathways is required, with particular emphasis on identifying high-impact components that drive postoperative recovery across different surgical settings. Such insights may inform more targeted and pragmatic approaches to ERAS implementation, especially in resource-constrained environments where comprehensive protocol adherence may not be feasible. The objective of this systematic review was to evaluate the association between individual components of ERAS protocols and postoperative outcomes across surgical specialties, and to identify key elements that are consistently associated with improved recovery.

## Review

Materials and methods

Study Design and Objective

This systematic review was conducted in accordance with the Preferred Reporting Items for Systematic Reviews and Meta-Analyses (PRISMA) 2020 guidelines [10]. The objective was to evaluate the association between individual components of enhanced recovery after surgery (ERAS) protocols and postoperative outcomes across surgical specialties, with a particular focus on identifying high-impact elements.

Study Design and PICO Framework

The eligibility criteria were defined using the Population, Intervention, Comparator, Outcomes, and Study Design (PICO) framework [11]. The population included adult patients undergoing elective surgical procedures across any specialty where ERAS protocols were implemented. The intervention was the application of ERAS pathways, either as complete protocols or through measurable adherence to individual components. Comparators included varying levels of ERAS compliance, absence of ERAS protocols, or differential adherence to specific ERAS elements. The primary outcomes of interest were postoperative length of stay and complication rates, while secondary outcomes included readmissions, mortality, and reoperation rates. Only observational cohort studies (prospective or retrospective) were included to ensure real-world applicability and consistency in study design.

Search Strategy and Information Sources

A comprehensive literature search was conducted across three electronic databases: PubMed/MEDLINE, Scopus, and Web of Science. The search covered studies published from January 1, 2015, to December 31, 2025, reflecting contemporary ERAS practices. The final search was performed on January 5, 2026.

Medical Subject Headings (MeSH) and free-text terms were used in combination with Boolean operators. The core search strategy included: (“Enhanced Recovery After Surgery” OR “ERAS” OR “fast-track surgery”) AND (“postoperative outcomes” OR “length of stay” OR “complications” OR “morbidity”) AND (“compliance” OR “adherence” OR “protocol elements” OR “components”)

MeSH terms such as “Enhanced Recovery After Surgery”, “Postoperative Complications”, and “Length of Stay” were incorporated where applicable. Boolean operators (“AND”, “OR”) and truncation strategies were used to maximize the sensitivity and specificity of the search. Reference lists of included studies were also screened to identify additional relevant articles.

Eligibility Criteria

Studies were included if they: (1) evaluated ERAS protocols in adult surgical populations; (2) reported overall ERAS compliance or the effects of individual ERAS components; (3) assessed at least one postoperative outcome, including length of stay, complications, or readmissions; and (4) were designed as prospective or retrospective cohort studies. Only studies providing sufficient detail on ERAS implementation or adherence to enable meaningful data extraction were considered.

Studies were excluded if they: (1) involved pediatric populations; (2) were randomized controlled trials, reviews, editorials, or case reports; (3) lacked clearly defined outcome measures; (4) did not report ERAS compliance or component-level data; or (5) were non-English. Randomized controlled trials were excluded as the objective was to assess real-world ERAS implementation and compliance, which are more appropriately captured in observational designs. Studies describing ERAS protocols without quantifiable adherence or component-level assessment were also excluded to maintain analytical rigor.

Study Selection

All retrieved records were screened independently based on titles and abstracts, followed by full-text evaluation for eligibility. Studies meeting the inclusion criteria were selected for qualitative synthesis. Discrepancies in study selection were resolved through consensus. The selection process was guided by PRISMA methodology to ensure transparency and reproducibility.

Data Extraction

Data were extracted using a predefined structured format, including study characteristics (author, year, surgical specialty, study design, sample size), ERAS protocol details, compliance metrics, individual components evaluated, and reported outcomes. Particular emphasis was placed on identifying components associated with multiple outcomes and consistency across studies.

Quality Assessment

Risk of bias for included studies was assessed using the Newcastle-Ottawa Scale (NOS) for cohort studies, evaluating domains of selection, comparability, and outcome assessment [12]. Studies were categorized as low, moderate, or high risk of bias based on NOS scoring. Given the observational nature of the included studies, particular attention was paid to potential confounding and selection bias.

Results

Study Selection Process

**Figure 1:**
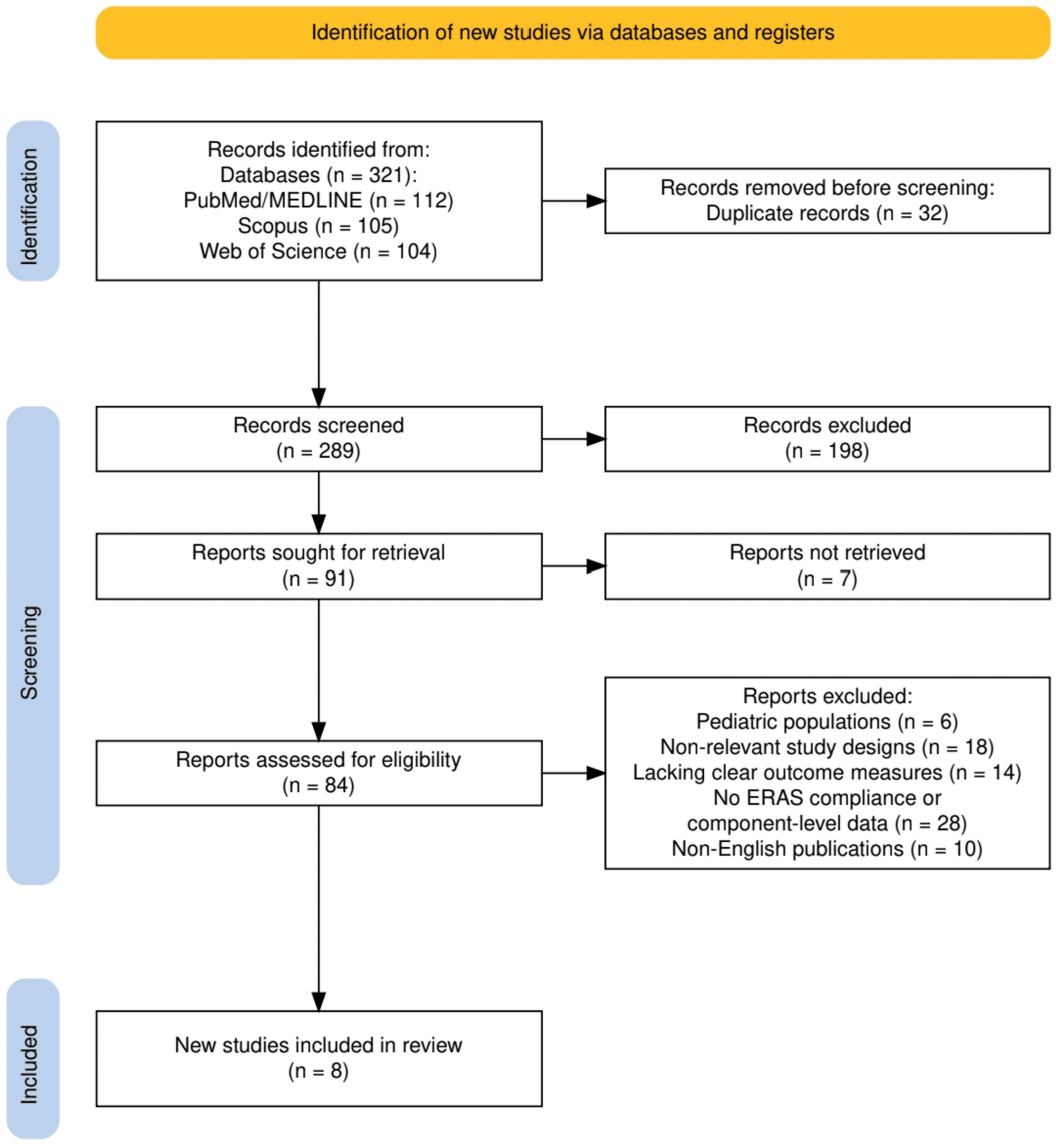
PRISMA flow diagram illustrating the study selection process for the systematic review PRISMA: Preferred Reporting Items for Systematic Reviews and Meta-Analyses

A total of 321 records were identified through database searching, including 112 from PubMed/MEDLINE, 105 from Scopus, and 104 from Web of Science. After removal of 32 duplicate records, 289 studies were screened based on titles and abstracts, of which 198 were excluded. Full-text assessment was conducted for 84 studies after excluding seven reports that could not be retrieved. Among these, 76 studies were excluded due to predefined criteria, including pediatric populations (n = 6), non-relevant study designs (n = 18), lack of clear outcome measures (n = 14), absence of ERAS compliance or component-level data (n = 28), and non-English publications (n = 10). Ultimately, eight studies met the inclusion criteria and were incorporated into the final qualitative synthesis. The detailed study selection process is illustrated in Figure 1.

Characteristics of the Selected Studies

The eight studies included in this review comprised a total of 4,959 patients and represented a broad spectrum of surgical specialties, including colorectal, hepatobiliary, pancreatic, thoracic, gynecologic, gastric, and head and neck surgery. Study designs were predominantly observational cohort studies, with a mix of prospective and retrospective methodologies. Sample sizes varied considerably, ranging from 210 to 1,175 patients, reflecting both single-center and multicenter experiences. The ERAS protocols evaluated across studies differed in both composition and reporting, with the number of components ranging from five to over twenty-seven elements. While some studies focused primarily on overall ERAS compliance, others provided more granular analyses of individual components and their association with outcomes. The most commonly assessed outcomes included postoperative length of stay and complication rates, with several studies also reporting on readmissions, mortality, and reoperation rates. Overall, the included studies demonstrated substantial heterogeneity in protocol structure, compliance measurement, and outcome reporting, which is summarized in Table 1.

**Table 1:** Characteristics of included studies evaluating ERAS components and postoperative outcomes ERAS: enhanced recovery after surgery; N: number of patients; NGT: nasogastric tube; IV: intravenous; LOS: length of stay; ML: machine learning; POD: postoperative day; ICU: intensive care unit

Study (year)	Surgery type	Study design/N	ERAS components evaluated	Key significant components	Outcomes assessed	Key findings
Jones et al. (2024) [13]	Colorectal	Retrospective cohort (n=1175)	Comprehensive ERAS (~14 components)	No NGT, early mobilization, early diet, multimodal analgesia, early IV fluid discontinuation	LOS, complications, readmission	Multiple components associated with improved outcomes; several impacted multiple endpoints; ML confirmed key predictors
Feng et al. (2022) [14]	Liver	Prospective cohort (n=436)	20 components (compliance-based)	Not analyzed individually	Major complications, LOS, readmission, reoperation	Higher compliance (>75–80%) associated with reduced complications and LOS; dose–response relationship
Burchard et al. (2022) [15]	Liver	Retrospective cohort (n=210)	Postoperative components (POD3)	Early diet, ambulation, drain removal, multimodal/oral analgesia, fluid discontinuation	LOS, complications	Early postoperative adherence associated with improved outcomes; ≥6 components by POD3 predictive
Iniesta et al. (2019) [16]	Gynecologic	Retrospective cohort (n=584)	ERAS (compliance + selected components)	Early mobilization, early nutrition, early Foley removal, fluid restriction	LOS, complications, readmission, reoperation	≥80% compliance associated with improved outcomes; select components reduced complications
Ritter et al. (2024) [17]	Pancreatic	Retrospective cohort (n=594)	27 components (compliance + individual)	Early mobilization (POD1), restrictive fluids, early catheter removal	Morbidity, mortality, LOS	High compliance improved outcomes; specific components independently predictive
Wagoner et al. (2025) [18]	Head & Neck	Retrospective cohort (n=257)	17 components (compliance-based)	Not individually analyzed	LOS, complications, readmission, ICU admission	Higher compliance improved outcomes; marked postoperative compliance gaps (mobilization)
Stuart et al. (2024) [19]	Thoracic	Prospective cohort (n=960)	5 components (compliance-based)	Not individually isolated	Morbidity, LOS	High compliance associated with reduced morbidity and LOS; strong dose–response effect
Ripollés-Melchor et al. (2023) [20]	Gastric	Prospective multicenter cohort (n=743)	22 components (adherence-based)	Not identified	Complications, LOS, mortality	No association between ERAS adherence and outcomes; highlights implementation variability

Quality Assessment

The methodological quality of the included studies was assessed using the Newcastle-Ottawa Scale for cohort studies, with most studies demonstrating low to moderate risk of bias. Prospective and multicenter cohorts generally achieved higher scores due to stronger study design, clearer outcome assessment, and greater external validity. Retrospective studies exhibited a moderate risk of bias, primarily related to potential selection bias and residual confounding. Studies incorporating multivariable analyses showed improved comparability despite inherent design limitations. Across the dataset, outcome assessment was largely reliable, given the use of objective clinical endpoints such as length of stay and complication rates. However, variability in ERAS protocol composition and compliance measurement introduced heterogeneity across studies. Overall, the included evidence was of moderate methodological quality, with key domains of bias summarized in Table 2.

**Table 2:** Quality assessment of included studies using the Newcastle-Ottawa Scale RoB: risk of bias; NOS: Newcastle-Ottawa Scale

Study (year)	Study design	Suitable RoB tool	Likely NOS rating	Overall risk of bias	Brief justification
Jones et al. (2024) [13]	Retrospective cohort	NOS	7–8/9	Moderate	Large sample, objective outcomes, multivariable analysis; limited by retrospective single-center design and possible residual confounding
Feng et al. (2022) [14]	Prospective cohort	NOS	8/9	Low to moderate	Prospective design, defined compliance groups, clear outcomes; still observational and vulnerable to confounding by care quality and case selection
Burchard et al. (2022) [15]	Retrospective cohort	NOS	7/9	Moderate	Useful component-level postoperative analysis, but retrospective design, smaller sample, and possible selection/performance bias
Iniesta et al. (2019) [16]	Retrospective cohort	NOS	7/9	Moderate	Reasonable cohort size and outcome assessment; compliance-based grouping and retrospective design introduce confounding risk
Ritter et al. (2024) [17]	Retrospective cohort	NOS	8/9	Low to moderate	Large sample, multivariable analysis, component-level assessment; retrospective single-center nature remains a limitation
Wagoner et al. (2025) [18]	Retrospective cohort	NOS	7/9	Moderate	Clear compliance framework and adjusted models; modest sample size and observational design increase confounding risk
Stuart et al. (2024) [19]	Prospective cohort	NOS	8/9	Low to moderate	Prospective multi-hospital design is a major strength; comparison with lower-compliance and historical control still allows bias from temporal and care-process differences
Ripollés-Melchor et al. (2023) [20]	Prospective multicenter cohort	NOS	8/9	Low to moderate	Strong external validity and prospective design; however, nonrandomized real-world adherence analysis may dilute effects and introduce center-level confounding

Discussion

Opening Synthesis

The present synthesis demonstrates that while enhanced recovery after surgery (ERAS) protocols are consistently associated with improved postoperative outcomes across diverse surgical specialties, the magnitude and consistency of these benefits appear to vary according to both the degree of protocol adherence and the specific components implemented. Across colorectal, hepatobiliary, pancreatic, thoracic, gynecologic, and head and neck procedures, higher compliance with ERAS pathways was generally associated with reductions in length of stay and postoperative morbidity, as evidenced in studies such as Feng et al., Ritter et al., and Stuart et al. [14,17,19]. However, this relationship was not uniformly observed, with large multicenter data from Ripollés-Melchor et al. demonstrating no significant improvement in outcomes with partial ERAS implementation, underscoring the heterogeneity of real-world practice [20]. Importantly, beyond overall compliance, several studies, most notably Jones et al. and Burchard et al., provide evidence that individual ERAS components exert differential effects on outcomes, suggesting that the benefits of ERAS may not derive from the protocol as a uniform bundle but rather from the effective execution of key elements within it [13,15].

Component-Level Insights

A central contribution of this review lies in the identification of specific ERAS components that demonstrate consistent associations with improved postoperative outcomes across multiple surgical domains. Component-level analyses, particularly those conducted by Jones et al. and Ritter et al., indicate that a subset of postoperative interventions, including early mobilization, early initiation of oral intake, optimization of perioperative fluid management, minimization of invasive tubes and catheters, and the use of multimodal analgesia, are repeatedly linked to reductions in complications and hospital length of stay [13,17]. These findings are further supported by Burchard et al., who emphasize the importance of early postoperative adherence to such elements within the initial recovery window [15]. The recurrence of these components across heterogeneous surgical populations suggests that they may reflect shared physiological mechanisms underlying recovery, rather than procedure-specific effects. Collectively, these observations support the concept that ERAS protocols may be functionally driven by a core group of high-yield interventions, with other components contributing variably depending on context and implementation fidelity [21].

Compliance Versus Individual Components

An important conceptual distinction emerging from this review is the relative contribution of overall ERAS compliance compared to the effect of individual protocol components. Several studies, including Feng et al., Wagoner et al., and Stuart et al., demonstrate a clear association between higher compliance rates and improved postoperative outcomes, suggesting that adherence to ERAS pathways as a whole is beneficial [14,18,19]. However, these compliance-based analyses do not elucidate which elements within the protocol are most influential. In contrast, component-level investigations by Jones et al., Burchard et al., and Ritter et al. indicate that certain interventions exert disproportionate effects on recovery [13,15,17]. Importantly, the observed relationship between compliance and outcomes may also reflect broader dimensions of perioperative care quality, including institutional experience, multidisciplinary coordination, and adherence to standardized care pathways, rather than the isolated effect of ERAS elements alone. As such, ERAS compliance may function as a composite surrogate marker, capturing both the consistent implementation of high-impact components and the overall quality of care delivery. This distinction is clinically relevant, as it supports a more targeted and context-sensitive approach to ERAS optimization, particularly in settings where full protocol adherence may be challenging [22].

Timing of ERAS Interventions

The temporal dimension of ERAS implementation represents a critical yet underexplored determinant of postoperative outcomes. Evidence from Burchard et al. highlights that adherence to key ERAS components within the early postoperative period, particularly by postoperative day three, is strongly associated with reduced length of stay and complication rates [15]. Similarly, Ritter et al. identify early mobilization on postoperative day one as an independent predictor of lower morbidity following pancreatic surgery [17]. These findings suggest that the early postoperative phase constitutes a pivotal window during which physiological recovery trajectories may be most modifiable. Interventions such as early ambulation, nutritional advancement, and timely fluid optimization likely exert their benefits by attenuating surgical stress responses and preventing secondary complications [23]. Consequently, the timing of ERAS component delivery may be as important as their selection, underscoring the need for protocols that prioritize early, coordinated postoperative care.

Cross-Specialty Consistency

The consistency of findings across diverse surgical disciplines represents a notable strength of the current evidence base and supports the broader generalizability of key ERAS principles. Despite variations in procedural complexity and perioperative pathways, studies spanning colorectal, hepatobiliary, pancreatic, thoracic, gynecologic, and head and neck surgery demonstrate convergence in both the direction and nature of outcomes associated with ERAS implementation. In particular, recurring associations between early mobilization, early enteral nutrition, and optimized fluid management with improved recovery metrics are observed across multiple cohorts, including those reported by Jones et al., Burchard et al., Iniesta et al., and Ritter et al. [13,15-17]. This cross-specialty reproducibility suggests that these interventions may act through shared physiological mechanisms, such as modulation of inflammatory responses, preservation of functional capacity, and prevention of postoperative complications. While procedure-specific adaptations remain necessary, the presence of these common elements indicates that a core set of ERAS components may be broadly applicable across surgical contexts.

Implementation Gaps

Notwithstanding the demonstrated benefits of ERAS pathways, substantial variability in real-world implementation remains a persistent challenge. Evidence from Wagoner et al. highlights marked discrepancies in compliance across perioperative phases, with notably low adherence to postoperative components such as early mobilization, despite high compliance with preoperative measures [18]. Similarly, the multicenter findings of Ripollés-Melchor et al. indicate that partial adoption of ERAS protocols or treatment within self-designated ERAS centers does not necessarily translate into improved outcomes, underscoring the limitations of incomplete or inconsistent implementation [20]. These observations suggest that the effectiveness of ERAS is highly dependent on the fidelity of execution, particularly in the postoperative period, where several high-impact components are concentrated [24]. Factors such as institutional resources, multidisciplinary coordination, and clinician adherence likely influence these gaps. Addressing these barriers through targeted implementation strategies and continuous compliance monitoring may be essential to fully realize the benefits of ERAS protocols in routine clinical practice.

Negative and Heterogeneous Findings

The interpretation of ERAS effectiveness must be contextualized within the presence of heterogeneous and, at times, discordant findings across the literature. While most studies demonstrate favorable associations between ERAS adherence and postoperative outcomes, the large multicenter analysis by Ripollés-Melchor et al. did not observe significant reductions in complications or length of stay with higher adherence levels or treatment within self-designated ERAS centers [20]. Such findings underscore the variability inherent in real-world implementation, including differences in protocol composition, institutional practices, and thresholds for adherence. Moreover, heterogeneity in outcome definitions and reporting standards across studies further complicates direct comparisons. It is also plausible that partial adherence to ERAS pathways fails to capture the synergistic effects of coordinated perioperative care, thereby attenuating measurable benefits. These considerations highlight the need for cautious interpretation and reinforce that ERAS effectiveness is not uniform but contingent upon both the quality and completeness of implementation [25].

Clinical Implications

The findings of this review carry several important implications for clinical practice, particularly in guiding the optimization and implementation of ERAS pathways. First, the consistent association between higher compliance and improved outcomes supports the continued emphasis on adherence monitoring as a key quality metric. However, the identification of specific high-impact components, including early mobilization, early enteral nutrition, and perioperative fluid optimization, suggests that prioritizing these elements may yield meaningful benefits even in settings where full protocol implementation is not feasible [26,27]. This is particularly relevant for resource-limited environments, where streamlined, evidence-informed ERAS models may enhance feasibility without substantially compromising efficacy. Furthermore, the importance of early postoperative interventions indicates that targeted efforts during the immediate recovery phase may have disproportionate clinical impact. Collectively, these insights support a more pragmatic and focused approach to ERAS implementation, emphasizing both adherence and strategic prioritization of key components to optimize patient outcomes.

Limitations

Several limitations should be considered when interpreting the findings of this review. First, the relatively small number of included studies may limit the breadth of evidence and the generalizability of the findings across surgical settings. Second, all included studies were observational in design, which limits the ability to establish causal relationships between ERAS components and postoperative outcomes and introduces the potential for residual confounding. In particular, higher ERAS compliance may reflect broader differences in institutional quality of care, multidisciplinary coordination, or patient selection rather than the isolated effect of specific protocol elements. Third, there was substantial heterogeneity across studies in terms of surgical specialties, ERAS protocol composition, and definitions of compliance and outcomes, which may limit comparability and generalizability of pooled interpretations. Fourth, detailed component-level analyses were available in only a subset of studies, with many investigations focusing primarily on overall compliance, thereby restricting the precision with which individual component effects can be delineated. Fifth, variability in reporting standards and adherence measurement may have introduced misclassification bias. Finally, the review protocol was not prospectively registered, which may introduce a risk of reporting bias despite adherence to established methodological guidelines. Despite these limitations, the inclusion of large, multicenter, and prospective cohorts enhances the overall robustness of the evidence base.

Future Directions

Future research should aim to refine the understanding of ERAS pathways by moving beyond aggregate compliance metrics toward more granular, component-specific evaluation. Well-designed prospective studies, and where feasible, randomized controlled trials, are needed to isolate the independent effects of individual ERAS interventions and to clarify their relative contributions to postoperative recovery. Standardization of ERAS reporting, including uniform definitions of adherence and outcome measures, would facilitate more meaningful comparisons across studies and settings. Additionally, the development of weighted or component-prioritized ERAS models may help optimize protocol design, particularly in resource-constrained environments. Emerging analytical approaches, including machine learning, may further aid in identifying high-impact components and interactions within complex perioperative pathways, as suggested by Jones et al. [13]. Finally, implementation science frameworks should be increasingly integrated into ERAS research to address barriers to adoption and to ensure that evidence-based protocols are translated effectively into routine clinical practice.

## Conclusions

This review demonstrates that adherence to enhanced recovery after surgery protocols is consistently associated with improved postoperative outcomes across multiple surgical specialties; however, these findings should be interpreted in the context of the observational design of the included studies and the substantial heterogeneity in surgical populations, ERAS protocols, and outcome definitions. The available evidence suggests that a subset of key interventions, particularly early mobilization, early enteral nutrition, optimization of perioperative fluid management, minimization of invasive tubes and catheters, and the use of multimodal analgesia, appear to contribute most significantly to improved recovery. At the same time, overall compliance remains an important determinant of outcomes, likely reflecting the consistent and coordinated delivery of these high-impact components as well as broader aspects of care quality. The variability observed across studies highlights that incomplete or inconsistent implementation may attenuate the effectiveness of ERAS pathways. Taken together, these findings support a more focused and pragmatic approach to ERAS, emphasizing both adherence and prioritization of core components, while underscoring the need for further high-quality, standardized, and prospective research to better delineate the independent effects of individual ERAS elements.
